# Secondary Effects of Hypochlorite Treatment on the Emerging Pollutant Candesartan: The Formation of Degradation Byproducts and Their Toxicological Profiles

**DOI:** 10.3390/molecules26113422

**Published:** 2021-06-05

**Authors:** Giovanni Luongo, Lorenzo Saviano, Giovanni Libralato, Marco Guida, Antonietta Siciliano, Lucio Previtera, Giovanni Di Fabio, Armando Zarrelli

**Affiliations:** 1 Department of Chemical Sciences, University of Naples Federico II, 80126 Naples, Italy; giovanni.luongo@unina.it (G.L.); difabio@unina.it (G.D.F.); 2 Department of Biology, University of Naples Federico II, 80126 Naples, Italy; lorenzosaviano@libero.it (L.S.); giovanni.libralato@unina.it (G.L.); marco.guida@unina.it (M.G.); antonietta.siciliano@unina.it (A.S.); 3 Associazione Italiana per la Promozione delle Ricerche su Ambiente e Salute umana, 82030 Dugenta, Italy; previter@unina.it

**Keywords:** candesartan, chlorination, hypochlorite, degradation byproducts, water treatment, battery toxicity test, *Daphnia magna*, *Aliivibrio fischeri*, *Raphidocelis subcapitata*

## Abstract

In recent years, many studies have reported the frequent detection of antihypertensive agents such as sartans (olmesartan, valsartan, irbesartan and candesartan) in the influents and effluents of wastewater treatment plants (WWTPs) and in the superficial waters of rivers and lakes in both Europe and North America. In this paper, the degradation pathway for candesartan (CAN) was investigated by simulating the chlorination process that is normally used to reduce microbial contamination in a WWTP. Twelve isolated degradation byproducts (DPs), four of which were isolated for the first time, were separated on a C-18 column by employing a gradient HPLC method, and their structures were identified by combining nuclear magnetic resonance and mass spectrometry and comparing the results with commercial standards. On the basis of these results, a mechanism of formation starting from the parent drug is proposed. The ecotoxicity of CAN and its DPs was studied by conducting a battery of ecotoxicity tests; bioassays were performed using *Aliivibrio fischeri* (bacterium), *Daphnia magna* (planktonic crustacean) and *Raphidocelis subcapitata* (alga). The ecotoxicity results shed new light on the increased toxicity of DPs compared with the parent compound.

## 1. Introduction

Emerging contaminants (ECs) have long been an object of attention for the scientific community and, more recently, by planning and control organizations at both global and national levels, and new substances or classes of substances are being increasingly identified and added to the lists of ECs [1,2,3,4,5,6]. ECs are chemical compounds that are found in very low concentrations (in the order of nanograms/L or a few micrograms/L at most or, more simply, as much as a sugar cube in a pond) in urban, agricultural and industrial waste and water bodies. Only a few years ago, it was possible to detect and measure them, and the development of increasingly complex and refined analytical tools has made it possible to quantify them, even at very low concentrations. Some examples of these compounds are the residues of pharmaceutical products for human and animal consumption, including drugs [7], pesticides [8], disinfectants [8], hormones and body care products [9]. These contaminants, even in very low quantities, can cause alterations to the endocrine system [3,10] and an increase in microbial resistance to drugs [11,12,13,14]. They can also be adsorbed by plants [15] and bioaccumulate [16] in the food chain. Additional risks are associated with biodiversity loss [10], infertility and cancer [17,18]. However, these ECs are not yet subject to regulatory control, and data relating to their environmental persistence and their chemical transformations in biotic and abiotic conditions are scarce, and even less information is known about their relative toxicology. Traditional wastewater treatment plants (WWTPs) have a limited capacity to remove many of these ECs because they have been designed and operate effectively to remove other types of pollutants (e.g., BOD, ammonia, phosphorus and pathogens) present in concentrations of up to a million times greater. It is not clear whether and which degradation byproducts (DPs) are obtained from the methods traditionally adopted in WWTPs before the waters return to the environment. For ECs whose effects and fate in the environment are still being studied, monitoring and research plans have been in place for several years, involving many institutions and research centers around the world. If these chemical compounds are regulated in the future, managers of integrated water services will have to adapt purification plants to effectively remove ECs and minimize or eliminate DPs.

One class of ECs is sartans (olmesartan, valsartan, irbesartan and candesartan), which are antihypertensive agents that act on the renin–angiotensin system with a different mechanism than ACE inhibitors; the latter reduces the plasma and tissue levels of angiotensin II, while sartans antagonize its action on AT1 receptors [19]. The attention of the scientific community has focused on this category of drugs, which have been detected in the aquatic environment [20]. In particular, candesartan (CAN) was found in Bavarian WWTP effluents at concentrations as high as 1712 ng/L [21]. Moreover, it is ubiquitous in England, where it was discovered at a concentration of 140 ng/L, and has been detected in surface waters at concentrations of up to 46 ng/L in Switzerland and up to 6.3 μg/L in Spain [22].

In this study, the DPs of CAN were investigated under the same conditions as those of the chlorination process normally used to reduce microbial contamination in WWTPs [23,24,25,26]. Two different experiments were performed: one at concentrations comparable to those at which CAN is present in wastewaters, and one at concentrations at least 100 times higher in order to isolate and identify the DPs. The structures of 12 isolated DPs, four of which were isolated for the first time, were determined by combining nuclear magnetic resonance (NMR) and mass spectrometry (MALDI-MS/TOF) data, and a mechanism of formation is proposed to explain the results.

The ecotoxicity of CAN before and after exposure to the chlorination process was evaluated. For this purpose, three living organisms, the bacteria *Aliivibrio fischeri*, the alga *Raphidocelis subcapitata* and the small planktonic crustacean *Daphnia magna*, were used as bioindicators of toxicity. These organisms were chosen because they are highly recommended as representative species for ecological risk assessments of new and existing chemicals [27]. The investigation on the toxicities of CAN and its byproducts is important for future discussions regarding the treatment, control and fate of sartans and their derivatives in the environment.

## 2. Results and Discussion

### 2.1. Chlorination Experiments 

**Figure 1:**
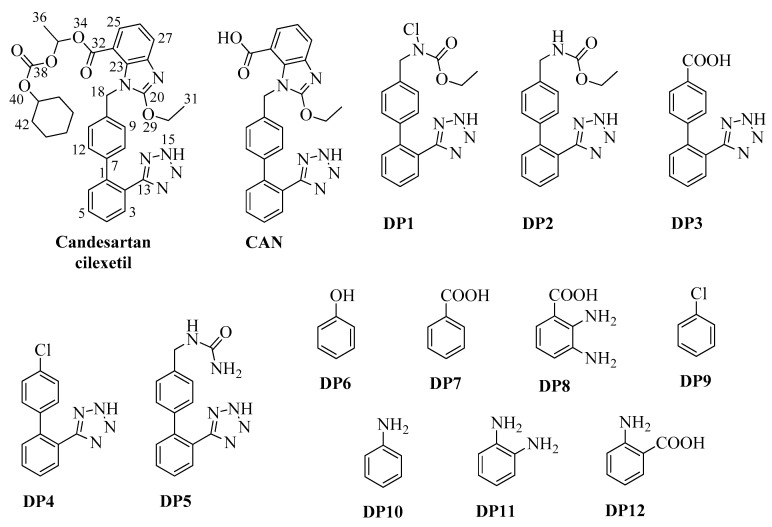
Chemical structures of Candesartan and its degradation byproducts.

The CAN chlorination experiments were performed by mimicking the conditions of a typical WWTP. A 10^−5^ M solution of the drug was treated for 1 h with 10% hypochlorite (CAN:hypochlorite molar ratio of 1:1; concn.) at room temperature [28,29,30,31]. Then, the tests were repeated with the contaminant at concentrations >10^−3^ M, with a much lower ratio of the CAN:oxidizing agent (1:5 or 1:6) so as to ensure the degradation of the studied contaminant and to isolate sufficient quantities of DPs for their subsequent structural identification. The DPs obtained (Figure 1) were isolated by column chromatography and HPLC and completely characterized using NMR and MS analyses (see Supplementary Materials).

**Figure 2:**
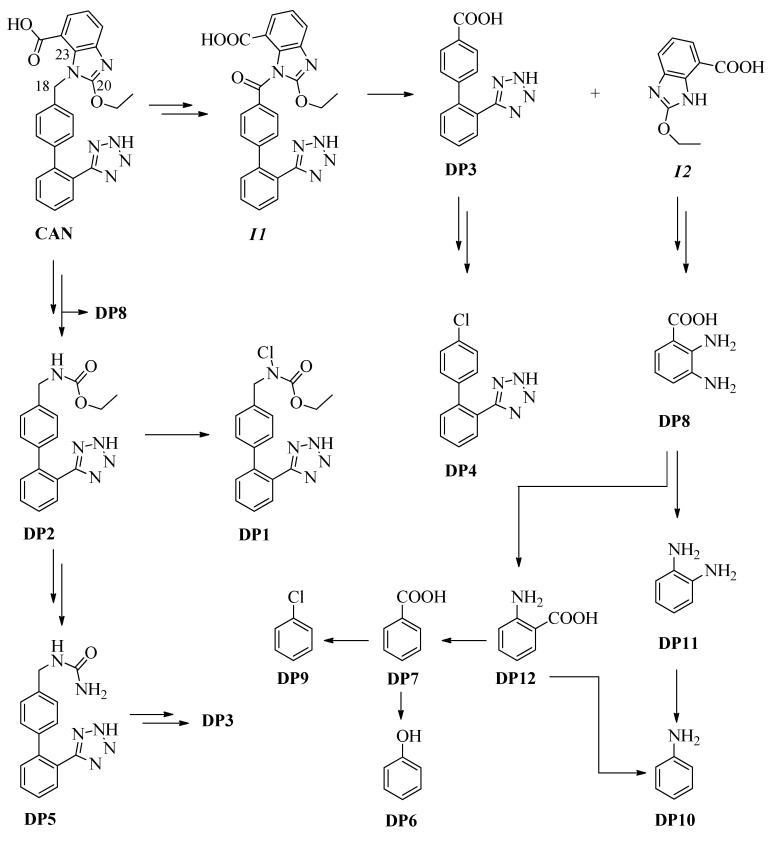
Plausible mechanism for the formation of **DP1**–**DP12**.

**DP1**–**DP12** were isolated in relative percentages of 2.4, 0.84, 0.64, 0.62, 1.62, 1.06, 1.55, 0.80, 0.42, 1.34, 0.62 and 0.86, respectively. The proposed mechanism of their formation from CAN is shown in Figure 2. **DP1**, **DP2**, **DP4** and **DP5** were isolated for the first time. The quantity of CAN recovered after chromatography of the initial extract of the chlorinated solution and its identification by comparison with an authentic commercial sample allowed us to evaluate a percentage of mineralization that is around 35% under the specified conditions, which is doubled with a CAN:hypochlorite molar ratio of 1:2 applied for longer times (3 h), while the formation of DPs is around 13%. The data reported in the literature for other ECs generally foresee longer reaction times, even in the order of hours, and oxidant concentrations are often double that of the pollutant to ensure the complete mineralization of the latter, frequently after a double or triple treatment [30,31,32,33].

### 2.2. Structure Elucidation of Degradation Byproducts **DP1**–**DP12**

**Scheme 1:**
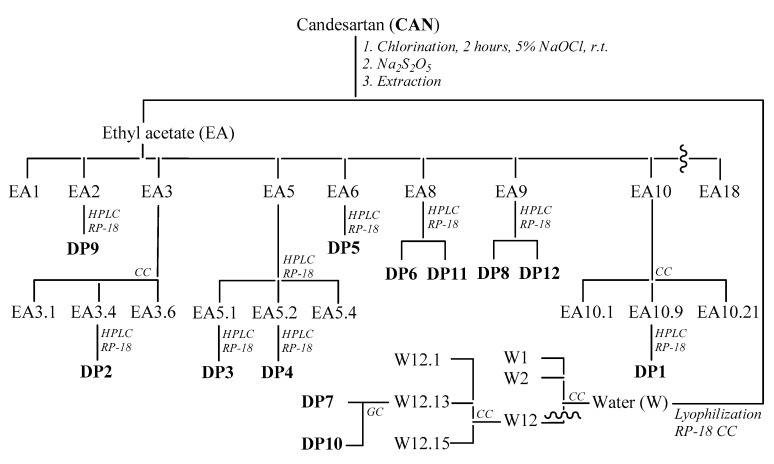
Isolation of the different identified compounds.

In CAN treatment at buffered pH, the changes of the drug were monitored by HPLC. The concentration of **DP1**–**DP12** (Figure 1) was at a maximum after 2 h and ranged in the range of 2.40 to 0.42%. They were isolated by chromatographic processes (Scheme 1) and identified by comparing their retention times with those of the standard compounds and by employing NMR and MS analyses. The plausible mechanism of the DPs formation from CAN is shown in Figure 2.

**DP1**–**DP5** derive from the loss of the benzo[*d*]imidazole ring, with the chain linked to carbon C10 chlorinated on N19 (**DP1**), non-chlorinated (**DP2**), oxidized to carboxyl (**DP3**), replaced by a chlorine (**DP4**) or engaged in the formation of a urethane derivative (**DP5**). **DP6**–**DP12** probably derive just from the degradation of the benzo[*d*]imidazole ring; in particular, we have three benzoic acids (**DP7**, **DP8** and **DP12**), aniline (**DP10**) and its derivative **DP11**, phenol (**DP6**) and chlorobenzene (**DP9**).

Under the conditions used, in accordance with the data reported in the literature [34,35,36,37], it is possible to hypothesize that CAN undergoes a first oxidation at carbon C18 to obtain the intermediate ***I1***. The latter could undergo the hydrolysis of the C18-N19 bond and release the product **DP3** and the intermediate ***I2***. **DP3** could first undergo a decarboxylation and then a subsequent chlorination to give **DP4**. Intermediate ***I2*** could undergo the hydrolysis of the N19-C20 and C20-N21 bonds to give **DP8**, from which **DP11** could then be obtained by decarboxylation and **DP12** by deamination. **DP10** could be obtained by deamination of **DP11** or decarboxylation of **DP12**. **DP12** by deamination could provide **DP7** and from the latter, by decarboxylation and subsequent oxidation, it could have **DP6** or, by decarboxylation and subsequent chlorination, **DP9**. The starting product for hydrolysis of the N19-C23 and C20-N21 bonds could release **DP2**, together with **DP8**, and from this one, obtain **DP1** by chlorination on N19. From **DP2** by reaction with ammonia, the final product of the complete degradation of the drug, it could produce urethane **DP5** and from this, by hydrolysis and subsequent oxidation, the product **DP3**. A plausible mechanism of the DPs formation from CAN is shown in Figure 2.

### 2.3. Ecotoxicity Data

**Figure 3:**
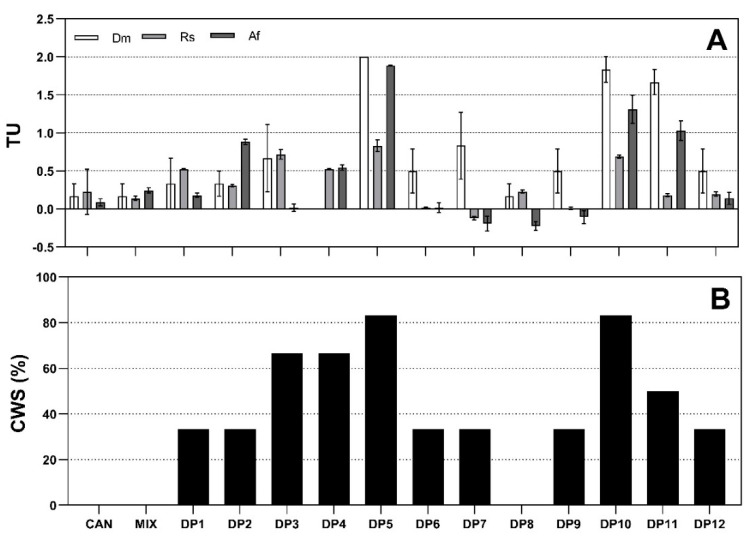
Toxic units (TU) for *D. magna* (Dm), *R. subcapitata* (Rs) and *A. fischeri* (Af) exposed to CAN and their DPs (**A**). Class weight scores (CWS %) for the selected battery tests (**B**).

Toxicity data were obtained using bioluminescent bacteria (*A. fischeri*), green algae (*R. subcapitata*) and crustaceans (*D. magna*). The results in Figure 3A reveal that both unicellular organisms were less sensitive than the multicellular species (*D. magna*), which unexpectedly proved to be the least resistant to the target compounds. The acute toxicity measured with aquatic organisms was expressed in toxic units (TU), where TU = 1/EC_50_. A class weight score (CWS) was calculated for each toxicity class in order to indicate the quantitative importance (weight) of the toxicity in that class [38]. Scores from the battery of toxicity tests performed on pharmaceutical samples were transformed into percentage values and assigned to surface water hazard categories (Figure 3B).

The results indicate that approximately 21.4% of samples (CAN, **DP8** and a mixture) are not a significant acute hazard (class I), while 42.9% of samples (**DP1**, **DP2**, **DP6**, **DP7**, **DP9** and **DP12**) are classified as a slight acute hazard (class II) according to the classification system. Of the remaining samples (**DP3**, **DP4**, **DP5**, **DP10** and **DP11**), 35.7% belong to the acute hazard category (class III) and can definitely pose serious risks to ecosystem integrity. Biostimulation was observed only in three samples (**DP7**, **DP8** and **DP9**), and the highest contribution was from the bacteria *A. fischeri*.

## 3. Materials and Methods

### 3.1. Drug and Reagents

Candesartan (99.5%) was purchased from Sigma Aldrich (Milan, Italy). All the other chemicals and solvents were purchased from Fluka (Saint-Quentin Fallavier, France) and were of HPLC grade and used as received. For the antimicrobial assessment, tryptic soy broth (TSB, Difco, Becton-Dickenson Labs, Heidelberg, Germany) was used. All chemicals were of analytical grade and supplied by Sigma Aldrich. Double-distilled water (Microtech, Naples, Italy) was used to prepare the dilution water and treatments. The microbial growth was measured with an automatic plate reader (Synergy HTX, BioTek Instruments, Winooski, VT, USA).

### 3.2. Chlorination Reaction

#### 3.2.1. Apparatus and Equipment

Column chromatography (CC) was carried out with Kieselgel 60 (230–400 mesh, Merck, Darmstadt, Germany). HPLC was performed on a Shimadzu LC-8A system using a Shimadzu SPD-10A VP UV-VIS detector (Shimadzu, Milan, Italy). Preparative HPLC was performed using an RP Gemini C18-110A preparative column (10 μm particle size, 250 mm × 21.2 mm i.d., Phenomenex, Bologna, Italy) with a flow rate of 7.0 mL/min. The ^1^H- and ^13^C-NMR spectra were recorded with an NMR spectrometer operated at 400 MHz and 25 °C (Bruker DRX, Bruker Avance, Billica, MA, USA) and referenced in ppm to the residual solvent signals (CDCl_3_, at δ_H_ 7.27 and δ_C_ 77.0). The proton-detected heteronuclear correlations were measured using a gradient heteronuclear single-quantum coherence (HSQC) experiment, optimized for ^1^J_HC_ = 155 Hz, and a gradient heteronuclear multiple bond coherence (HMBC) experiment, optimized for ^n^J_HC_ = 8 Hz. MALDI-TOF mass spectrometric analyses were performed on a Voyager-De Pro MALDI mass spectrometer (PerSeptive Biosystems, Framingham, MA, USA). The UV-VIS spectra were recorded with a Perkin Elmer Lambda 7 spectrophotometer. The IR spectra were recorded with a Jasco FT/IR-430 instrument equipped with a single reflection ATR accessory. A WTW pH 7110 pH meter (Xylem Analytics, Weilheim, Germany) equipped with a WTW SenTix41 electrode with a temperature sensor was used to record pH.

#### 3.2.2. Chlorination Experiments

A 10^−5^ M CAN solution was treated for 1 h with 10% hypochlorite (molar ratio CAN/HClO 1:1 concentration, spectroscopically determined at λ_max_ = 292 nm and ε 350 = dm^3^/mol cm) at room temperature [27], simulating the conditions used in a typical WWTP. The presence of CAN was quantified using the UV-VIS spectrophotometer. Absorbance peaks were determined at 230 nm. The absorbance values were converted into concentration using a calibration curve prepared from standard solutions with known CAN concentrations. The pH of the solution, measured and recorded continuously using a pH meter, increased immediately from the initial pH of 8.0 to 10.5, and the pH remained at this value during the reaction. An aliquot of the solution was quenched by excess sodium thiosulfate, filtered and dried by lyophilization. The residue was dissolved in a saturated sodium bicarbonate solution and extracted with ethyl acetate. The course of the reaction was monitored by HPLC. The main degradation byproducts (**DP1**–**DP6**, **DP8**–**DP9** and **DP11**–**DP12** for the ethyl acetate fraction and **DP7** and **DP10** for the aqueous fraction; Scheme 1 and Figure 1) were identified by comparing their retention times with those of commercially available standard compounds or those isolated by performing preparative experiments with a solution of CAN at a concentration higher than 10^−3^ M treated with 5% hypochlorite (molar ratio CAN/HClO 1:20 concentration) at room temperature for 2 h. The DPs obtained were isolated by column chromatography and HPLC and completely characterized using NMR and MS analyses.

#### 3.2.3. Chlorination Procedure and Product Isolation

Candesartan (0.5 g, 1.14 mmol) was dissolved in phosphate buffer (KH_2_PO_4_/K_2_HPO_4_ 0.1 M, 500 mL), and 5% hypochlorite was added drop by drop (molar ratio CAN/HClO 1:20; concentration spectroscopically determined at λ_max_ = 292 nm, ε = 350 dm^3^/mol cm) at room temperature [28]. The pH of the solution, monitored with a pH meter, was adjusted by adding a 10% H_3_PO_4_ solution, and it remained stable at 6.5 for the entire duration of the experiment. The solution was quenched after 2 h with an excess of sodium thiosulfate, concentrated by lyophilization and extracted with ethyl acetate (EA) and water (W). The crude EA fraction (446 mg) was chromatographed on silica gel CC, eluting with a gradient of methylene chloride:methanol:acetic acid (100:0:0.5 to 70:30:0.5, *v*/*v*/*v*) to yield 18 fractions.

Fraction EA2 (9 mg), eluted with methylene chloride:methanol:acetic acid (100:0:0.5, *v*/*v*/*v*), was analyzed by HPLC using a reversed-phase column (Luna 5 µm 100 Å C18(2); 150 × 4.6 mm) and eluting with water:methanol (20:80, *v*/*v*) at a solvent flow rate of 1 mL/min to yield **DP9** (*t*_R_ 7.2 min, 2.1 mg).

Fraction EA3 (47 mg), eluted with methylene chloride:methanol:acetic acid (98:2:0.5, *v*/*v*/*v*), was re-chromatographed on silica gel CC by eluting with a gradient of petrol ether:acetone (100:0 to 50:50, *v*/*v*) to yield 6 subfractions. Subfraction EA3.4 (13 mg), eluted with petrol ether:acetone (50:50, *v*/*v*), was analyzed by HPLC using a reversed-phase column (Kromasil 10 µm 100 Å C18; 250 × 10 mm) and eluting with a gradient of CH_3_COONH_4_ (A, pH 4.0; 10 mM) and methanol (B), starting with 70% B for 1 min and establishing a gradient to obtain 100% B over 20 min at a solvent flow rate of 4 mL/min, in order to yield **DP2** (*t*_R_ 17.9 min, 4.2 mg).

Fraction EA5 (57 mg), eluted with methylene chloride:methanol:acetic acid (90:10:0.5, *v*/*v*/*v*), was separated by semipreparative HPLC using a reversed-phase column (Kromasil 10 µm 100 Å C18; 250 × 10 mm) and eluting with a gradient of CH_3_COONH_4_ (A, pH 4.0; 10 mM) and methanol (B), starting with 60% B for 1 min and establishing a gradient to obtain 100% B over 30 min at a solvent flow rate of 4 mL/min, in order to yield 4 subfractions. Subfractions EA5.1 (8 mg) and EA5.2 (5 mg) were re-chromatographed by HPLC using a reversed-phase column (Kinetex 2.6 µm 100 Å C18; 100 × 4.6 mm) and eluting with a gradient of acetic acid:methanol (A, 1:99, *v*/*v*) and acetic acid:water (B, 1:99, *v*/*v*), starting with 60% B for 5 min and establishing a gradient to obtain 100% A over 35 min and returning to 60% B for 10 min at a solvent flow rate of 0.8 mL/min. They contained **DP3** (*t*_R_ 9.8 min, 3.2 mg) and **DP4** (*t*_R_ 17.3 min, 3.1 mg), respectively.

Fraction EA6 (11 mg), eluted with methylene chloride:methanol:acetic acid (85:15:0.5, *v*/*v*/*v*), was analyzed by HPLC using a reversed-phase column (Luna 5 µm 100 Å C18(2); 150 × 4.6 mm) and eluting with a gradient of CH_3_COONH_4_ (A, pH 4.0; 10 mM) and methanol (B), starting with 40% B for 1 min and establishing a gradient to obtain 100% B over 20 min at a solvent flow rate of 1 mL/min, in order to yield **DP5** (*t*_R_ 11.5 min, 8.1 mg).

Fraction EA8 (38 mg), eluted with methylene chloride:methanol:acetic acid (80:20:0.5, *v*/*v*/*v*), was analyzed by HPLC using a reversed phase column and eluting with a gradient of CH_3_COONH_4_ (A, pH 4.0; 10 mM) and acetonitrile (B), starting with 20% B for 1 min and establishing a gradient to obtain 90% B over 25 min at a solvent flow rate of 3.5 mL/min, in order to yield **DP6** (*t*_R_ 11.3 min, 5.3 mg) and **DP11** (*t*_R_ 15.0 min, 3.1 mg).

Fraction EA9 (19 mg), eluted with methylene chloride:methanol:acetic acid (75:25:0.5, *v*/*v*/*v*), was analyzed by HPLC using a reversed-phase column (Kromasil 10 µm 100 Å C18; 250 × 10 mm) and eluting with a gradient of water (A) and MeOH (B), starting with 10% A for 1 min and establishing a gradient to obtain 10% A over 30 min at a solvent flow rate of 1 mL/min, in order to yield **DP8** (*t*_R_ 5.3 min, 4.0 mg) and **DP12** (*t*_R_ 7.1 min, 4.3 mg).

Fraction EA10 (203 mg), eluted with methylene chloride:methanol:acetic acid (70:30:0.5, *v*/*v*/*v*), was re-chromatographed on silica gel CC by eluting with a gradient of chloroform:acetone (100:0 to 60:40, *v*/*v*) to yield 21 subfractions. Subfraction EA10.9 (73 mg), eluted with chloroform:acetone (70:30, *v*/*v*), was analyzed by preparative HPLC using a reversed-phase column (Gemini 10 µm C18 110 Å; 250 × 21 mm) and eluting with a gradient of CH_3_COONH_4_ (A, pH 4.0; 10 mM) and methanol (B), starting with 0% B for 1 min and establishing a gradient to obtain 100% B over 20 min at a solvent flow rate of 7.5 mL/min, in order to yield **DP1** (*t*_R_ 18.4 min, 12.0 mg). 

The aqueous fraction (W, 3.5 g) was dried by lyophilization, re-dissolved in water and filtered on an RP-18 reversed-phase silica gel for column chromatography (CC) using a gradient of water:methanol (100:0 to 0:100, *v*/*v*) to yield 12 fractions. The last fraction, eluted with methanol (165 mg), was dried by a rotary evaporator, re-dissolved in methanol and separated with silica gel CC using a gradient of methylene chloride:methanol (100:0 to 0:100, *v*/*v*) to yield 15 fractions. 

Fraction W12.13 (29 mg), eluted with methylene chloride:methanol (70:30, *v*/*v*), was dried, dissolved in an appropriate volume of methylene chloride (100 μL) and analyzed using a Shimadzu 2010 series GC FID (Shimadzu, Milano, Italy). The gas chromatograph was equipped with an Equity^TM^-5 capillary column (30 m × 0.25 mm I.D. × 0.5 μm film thickness). The following parameters were set during the experiments: a detector temperature of 325 °C, helium as the carrier gas (25 cm/s) and an injected sample volume of 1.0 μL, introduced into the injector using an AOC-20i autosampler (Shimadzu, Milano, Italy) heated to 280 °C with a split ratio of 100:1. The initial temperature was 40 °C (2 min), which was increased at 50 °C/min to 100 °C, 10 °C/min to 200 °C and 30 °C/min to 325 °C (7.5 min). **DP7** (*t*_R_ 15.9 min, 7.6 mg) and **DP10** (*t*_R_ 21.2 min, 6.7 mg) were identified by comparing the results with data on standard compounds and their relative calibration lines.

### 3.3. Spectral Data

Candesartan cilexetil: *1-(((cyclohexyloxy)carbonyl)oxy)ethyl 1-((2′-(2H-tetrazol-5- yl)-[1,1′-biphenyl]-4-yl)methyl)-2-ethoxy-1H-benzo[d]imidazole-7-carboxylate*. White powder. ^1^H- and ^13^C-NMR (see Table S1). MS-TOF (positive ions): *m*/*z* calculated for C_33_H_34_N_6_O_6_ *m*/*z* 610.25 [M]^+^; found 611.65 [M + H]^+^ (28%).

Candesartan: *1-((2′-(2H-tetrazol-5-yl)-[1,1′-biphenyl]-4-yl)methyl)-2-ethoxy-1H-ben zo[d]imidazole-7-carboxylic acid*. White powder. ^1^H- and ^13^C-NMR (see Table S2). MS-TOF (positive ions): *m*/*z* calculated for C_24_H_20_N_6_O_3_ *m*/*z* 440.16 [M]^+^; found 441.46 [M + H]^+^ (40%). 

**DP1**: *Ethyl ((2′-(2H-tetrazol-5-yl)-[1,1′-biphenyl]-4-yl)methyl)chlorocarbamate*. White powder. ^1^H- and ^13^C-NMR (see Table S3). MS-TOF (positive ions): *m*/*z* calculated for C_17_H_16_N_5_O_2_Cl *m*/*z* 357.10 [M]^+^ and 359.11 [M + 2]^+^; found 357.08 [M]^+^ (78%); 359.09 [M + 2]^+^ (37%).

**DP2**: *Ethyl ((2′-(2H-Tetrazol-5-yl)-[1,1′-biphenyl]-4-yl)methyl)carbamate.* White powder. ^1^H- and ^13^C-NMR (see Table S4). MS-TOF (positive ions): *m*/*z* calculated for C_17_H_17_N_5_O_2_ *m*/*z* 323.14 [M]^+^; found 324.37 [M + H]^+^ (63%).

**DP3**: *2′-(2H-Tetrazol-5-yl)-[1,1′-biphenyl]-4-carboxylic acid.* White powder. ^1^H- and ^13^C-NMR (see Table S5). MS-TOF (positive ions): *m*/*z* calculated for C_14_H_10_N_4_O_2_ *m*/*z* 266.08 [M]^+^; found 267.26 [M + H]^+^ (58%) [29].

**DP4**: *5-(4′-Chloro-[1,1′-biphenyl]-2-yl)-2H-tetrazole.* White powder. ^1^H- and ^13^C-NMR (see Table S6). MS-TOF (positive ions): *m*/*z* calculated for C_13_H_9_N_4_Cl *m*/*z* 256.05 [M]^+^ and 258.04 [M + 2]^+^; found 256.02 [M]^+^ (94%); 258.04 [M + 2]^+^ (43%).

**DP5**: *1-((2′-(2H-Tetrazol-5-yl)-[1,1′-biphenyl]-4-yl)methyl)urea.* White powder. ^1^H- and ^13^C-NMR (see Table S7). MS-TOF (positive ions): *m*/*z* calculated for C_15_H_14_N_6_O *m*/*z* 294.12 [M]^+^; found 295.22 [M + H]^+^ (45%).

**DP6**: *Phenol.* White oil. NMR spectra conform to those recorded for the commercially available standard [39].

**DP7**: *Benzoic acid.* Identified by comparison with standard compounds [39]. 

**DP8**: *2,3-Diaminobenzoic acid*. White powder. NMR spectra conform to those recorded for the commercially available standard [40].

**DP9**: *Chlorobenzene.* White oil. NMR spectra conform to those recorded for the commercially available standard [39].

**DP10**: *Aniline.* Identified by comparison with an authentic sample [39]. 

**DP11**: *1,2-Benzenediamine.* White powder. NMR spectra are in accordance with those reported in the literature [39].

**DP12**: *2-Aminobenzoic acid.* White powder. NMR spectra conform to those recorded for the commercially available standard [39].

### 3.4. Ecotoxicity Tests

The toxicity of candesartan and its DPs was assessed towards three organisms, *A. fischeri*, *R. subcapitata* and *D. magna*.

The acute bioluminescence assay was detected with the *A. fischeri* (NRRLB-11177) bacterium supplied by MicroBioTest, Gent, Belgium, and activated by rehydration with a reconstitution solution, according to ISO 11348-3:2007 [41]. These toxicity experiments were carried out with a luminometer Microtox (Model 500 analyzer, New Castle, DE, USA), according to the international procedure (ISO 11348-3). To provide the required osmotic pressure for the test organism, the assay was conducted using a saline water solution (2% sodium chloride, NaCl). The bioluminescence intensity of *A. fischeri* bacteria was measured after 30 min at 490 nm of exposition to the solutions containing CAN and its DPs at 15 °C. The test was performed in triplicate with a control, and pharmaceutical and toxic effect values were expressed as the ratio of the decrease in bacterial light production to the remaining light. 

The algal growth inhibition test was performed according to the European standard EN ISO 8692:2012 [42] using *R. subcapitata*. Algal cells in the exponential growth phase were taken from an exponentially growing pre-culture according to ISO 8692 [37], and it was added to the solution of CAN and its DPs to reach a final algal concentration of 10^4^ cells/mL. Algal toxicity tests were carried out in three replicates and maintained under continuous illumination (white fluorescent light) at 22 ± 4 °C for incubation and were mixed twice a day by hand. The algal growth was measured after 72 h at 670 nm (HachLange DR 5000, Loveland, CO, USA). The difference between the growth rate in the control group and the solutions provided inhibition rate in each samples. 

The acute immobilization test with *D. magna* was performed according to the European Standard EN ISO 6341:2013 [43]. Daphnids (less than 24 h old) obtained from a laboratory culture were used (20 organisms for each pharmaceutical and control). In the acute assay, three parallel replicates of 6-well plates were used, and the number of immobilized organisms was determined under stereomicroscopy (LEICA EZ4-HD) after 24 and 48 h of exposure. Daphnids were considered immobilized if they lost the aptitude to perform vertical swimming after 15 s of observation and after slight agitation of the multiwell plate.

Toxicity of the samples was expressed as Toxic Unit (TU), where TU = 1/EC50. The homoscedasticity (F test, *p* < 0.05) and normality (Shapiro–Wilk test, *p* < 0.05) of data were verified using Prism 8 (GraphPad Inc., Version 8.4.2, San Diego, CA, USA). TU data were integrated according to Persoone et al.’s [38] approach for natural water and a class weight score (CWS) was determined by averaging the values corresponding to each microbiotest class normalized to the most sensitive organism (highest score).

## 4. Conclusions 

This study investigated the fate of candesartan by monitoring its degradation using an excess of sodium hypochlorite. After the chlorination treatment, chromatographic techniques were used to isolate 12 degradation byproducts, four of which were isolated for the first time, which were fully characterized by MS and NMR analyses and compared with parental samples. Some byproducts retain the diphenyl base nucleus and the tetrazole ring of the side chain, but undergo the partial degradation (**DP1**–**DP2** and **DP5**) or the loss (**DP3** and **DP4**) of benzimidazole; others have a C_6_C_1_ (**DP7**, **DP8** and **DP12**) or C_6_C_0_ (**DP6**, **DP9**–**DP11**) skeleton, obviously being the degradation products of the first five. About 35% of the initial quantity of candesartan is estimated to undergo complete mineralization, and about 13% is transformed into degradation byproducts. A possible mechanism for the degradation of candesartan and the formation of its degradation byproducts is proposed. The CWS approach confirmed that 78.6% of the total investigated pharmaceutical samples are able to have some adverse ecotoxicological effects on the aquatic environment and that effluents containing CAN degradation byproducts with pollutant potential could generate negative impacts on water and/or soil equilibrium, as well as human health. It would be interesting in the future to use the waters of a WWTP to consider, for example, the effect of ammonia present (or other pollutants) as well as to compare the byproducts of candesartan obtained by hypochlorite with those that could be obtained by ozonation or other oxidative degradation methods (UV, peracetic acid, …).

## Supplementary Materials

The following are available online, ^1^ H-, ^13^C- and 2D-NMR data of Candesartan Cilexetil, Candesartan and **DP1**–**DP5**, Tables S1–S7.

## Sample Availability

Samples of the compounds **DP6**–**DP12** are available from the authors.
