# Supplementary material for: Secondary Effects of Hypochlorite Treatment on the Emerging Pollutant Candesartan: The Formation of Degradation Byproducts and Their Toxicological Profiles
Source: Molecules. 2021 Jun 5;26(11):3422. doi: 10.3390/molecules26113422 (PMC8200957; doi:10.3390/molecules26113422)
Supplement: Supplementary file 1 [file molecules-26-03422-s001.zip › molecules-1211823-supplementary.pdf]

Supplementary Material

Secondary Effects of Hypochlorite Treatment on the Emerging Pollutant Candesartan: The Formation of Degradation Byproducts and Their Toxicological Profiles

Giovanni Luongo, Antonietta Siciliano, Marco Guida, Giovanni Libralato, Lorenzo Saviano, Lucio Previtera, Giovanni Di Fabio and Armando Zarrelli*

* Correspondence: zarrelli@unina.it; Tel.: +39-081-674-472

Table S1. ^{1}H, ^{13}C and 2D NMR data of Candesartan Cilexetil in $CDCl_3$.

Position	Residue	$^{13}C^a$	$^{1}H^a$ (J in Hz)	^{1}H-^{1}H COSY	^{1}H-^{13}C HMBC
1	C	140.80	-		6.90, 7.57, 8.01
2	C	123.08	-		7.33, 7.56
3	CH	131.27	8.01, dd (9.4, 3.7)	7.56	7.57
4	CH	128.26	7.56, t (8.0)	7.57, 8.01	7.33
5	CH	130.54	7.57, m	7.33, 7.56, 8.01	7.56, 8.01
6	CH	131.10	7.33, dd (9.1, 3.9)	7.56	7.56
7	C	138.00	-		6.80, 7.3
8/12	CH	129.39	6.90, d (8.4)	6.80	6.80, 6.90
9/11	CH	125.48	6.80, d (8.4)	6.90	6.80, 6.90
10	C	136.82	-		6.90
13	C	154.90	-		8.01
18	CH_2	46.93	5.61, d (15.9)<br>5.67, d (15.9)	5.67<br>5.61	6.80<br>6.80
20	C	158.07	-		4.51, 5.61, 5.67
22	C	140.32	-		7.02
23	C	130.92	-		7.17, 7.55
24	C	115.03	-		7.02
25	CH	124.26	7.55, m	7.02, 7.17	7.17
26	CH	121.21	7.02, t (7.6)	7.17, 7.55	7.17
27	CH	121.47	7.17, d (6.9)	7.02, 7.55	7.55
29	CH_2	67.53	4.51, m	1.44	4.51
30	CH_3	14.52	1.44, m	4.51	1.44
31	C	163.40	-		7.55
34	CH	91.90	6.72, q (5.2)	1.46	1.46
35	CH_3	19.26	1.46, d (5.2)	6.72	6.72
37	C	152.41	-		
40	CH	77.82	4.51, m	1.35, 1.81	1.35, 1.81
41/45	CH_2	31.27	1.35, m<br>1.81, m	1.20, 1.58, 4.51	1.20, 1.58, 4.51
42/44	CH_2	23.44	1.20, m<br>1.58, m	1.35, 1.81	1.47, 1.81
43	CH_2	24.95	1.47, t (7.0)	1.20, 1.58	1.58, 1.81

[a]Chemical shifts in ppm.

Table S2. ^{1}H, ^{13}C and 2D NMR data of Candesartan in $CDCl_3/CD_3OD$ 9:1.

Position	Residue	$^{13}C^{a}$	$^{1}H^{a}$ (J in Hz)	^{1}H-^{1}H COSY	^{1}H-^{13}C HMBC
1	C	141.03	-		6.98, 7.55, 7.64
2	C	122.27	-		7.42, 7.46
3	CH	130.38	7.64, d (7.8)	7.46, 7.55	7.55
4	CH	127.51	7.46, t (7.6, 1.0)	7.55, 7.64	7.42, 7.55
5	CH	130.19	7.55, t (7.8, 1.4)	7.42, 7.46, 7.64	7.46, 7.64
6	CH	130.93	7.42, d (7.6, 1.2)	7.46, 7.55	7.46
7	C	137.74	-		6.93, 7.42
8/12	CH	128.74	6.98, d (8.4)	6.93	6.93, 6.98
9/11	CH	126.48	6.93, d (8.4)	6.98	6.93, 6.98
10	C	136.63	-		6.98
13	C	158.20	-		7.64
18	CH_2	46.66	5.61, s		693
20	C	154.90	-		4.55, 5.61
22	C	140.67	-		7.16
23	C	130.84	-		7.64
24	C	116.19	-		7.16
25	CH	121.01	7.65, d (7.8, 1.1)	7.16, 7.64	7.64
26	CH	124.04	7.16, t (8.0)	7.64	7.64
27	CH	120.78	7.65, d (7.8, 1.1)	7.16	7.64
29	CH_2	66.62	4.55, q (7.1)	1.43	1.43
30	CH_3	13.92	1.43, t (7.1)	4.55	4.55
31	C	168.15	-		7.64

[a]Chemical shifts in ppm.

Table S3. ^{1}H, ^{13}C and 2D NMR data of **DP1** in CD_3OD.

Position	Residue	$^{13}C^{a}$	$^{1}H^{a}$ (J in Hz)	^{1}H-^{1}H COSY	^{1}H-^{13}C HMBC
1	C	141.50	-		7.02, 7.50, 7.52
2	C	123.55	-		7.47, 7.49
3	CH	130.34	7.52, d (7.3)	7.49, 7.50	7.50
4	CH	127.33	7.49, dd (7.7, 1.1)	7.47, 7.50, 7.52	7.47, 7.50
5	CH	130.27	7.50, dd (7.7, 1.3)	7.47, 7.49, 7.52	7.47, 7.52
6	CH	130.74	7.47, d (8.1)	7.49, 7.50	7.49
7	C	138.84	-		7.16, 7.47
8/12	CH	128.84	7.02, d (8.0)	7.16	7.02, 7.16
9/11	CH	127.48	7.16, d (8.0)	7.02	7.02, 7.16
10	C	135.86	-		7.02
13	C	155.94	-		7.52
18	CH_2	45.59	5.28, s		7.16
20	C	159.16	-		4.55
29	CH_2	67.23	4.55, q (7.1)	1.35	1.35
30	CH_3	13.40	1.35, t (7.1)	4.55	4.55

[a]Chemical shifts in ppm.

Table S4. [1] H, ^{13}C and 2D NMR data of **DP2** in CD_3OD.

Position	Residue	$^{13}C^a$	$^1H^a$ (J in Hz)	^1H-^1H COSY	^1H-^{13}C HMBC
1	C	143.27	-		7.63
2	C	125.62	-		7.53
3	CH	132.07	7.63, m	7.53	7.53, 7.63
4	CH	127.83	7.53, m	7.63	7.53, 7.63
5	CH	131.67	7.63, m	7.53	7.53, 7.63
6	CH	130.22	7.53, m	7.63	7.53, 7.63
7	C	139.74	-		7.21, 7.53
8/12	CH	129.96	7.07, d (8.5)	7.21	7.07, 7.21
9/11	CH	128.87	7.21, d (8.5)	7.07	7.07, 7.21
10	C	140.26	-		7.07
13	C	157.86	-		7.63
18	CH_2	44.99	4.25, s		7.21
20	C	158.85	-		4.50
29	CH_2	65.65	4.08, q (7.2)	1.28	1.28
30	CH_3	13.01	1.28, t (7.1)	4.50	4.50

[a]Chemical shifts in ppm.

Table S5. ^{1}H, ^{13}C and 2D NMR data of **DP3** in CD_3OD.

Position	Residue	$^{13}C^a$	$^{1}H^a$ (J in Hz)	^{1}H-^{1}H COSY	^{1}H-^{13}C HMBC
1	C	142.71	-		7.51, 7.57
2	C	134.06	-		7.45, 7.48
3	CH	131.81	7.57, dd (7.5, 1.2)	7.45, 7.51	7.51
4	CH	128.46	7.45, dt (7.4, 1.9)	7.48, 7.51, 7.57	7.48, 7.51
5	CH	131.20	7.51, dt (7.1, 1.4)	7.45, 7.48, 7.57	7.57
6	CH	130.33	7.48, dd (7.1, 1.3)	7.45, 7.51	7.45
7	C	145.81	-		7.48, 7.81
8/12	CH	130.00	7.15, d (8.6)	7.81	7.15, 7.81
9/11	CH	130.01	7.81, d (8.6)	7.15	7.15, 7.81
10	C	134.06	-		7.15
13	C	162.50	-		7.57
18	C	173.10	-		7.81

[a]Chemical shifts in ppm.

Table S6. [1] H, ^{13}C and 2D NMR data of **DP4** in CD_3OD.

Position	Residue	^{13}C[a]	^{1}H[a] (J in Hz)	^{1}H-^{1}H COSY	^{1}H-^{13}C HMBC
1	C	141.63	-		7.30, 7.66, 7.70
2	C	126.79	-		7.56, 7.59
3	CH	131.58	7.70, dd (7.5, 1.2)	7.59, 766	7.66
4	CH	129.74	7.59, dt (7.6, 1.6)	7.56, 7.66, 7.70	7.56, 7.66
5	CH	131.84	7.66, dt (7.6, 1.5)	7.56, 7.59	7.56, 7.59, 7.70
6	CH	131.23	7.56, dd (7.6, 1.2)	7.59, 7.66	7.59, 7.66
7	C	146.45	-		7.56, 7.65
8/12	CH	131.23	7.30, d (8.7)	7.65	7.30, 7.65
9/11	CH	133.10	7.65, d (8.7)	7.30	7.30, 7.65
10	C	119.61	-		7.30
13	C	158.85	-		7.70

[a]Chemical shifts in ppm.

Table S7. ^{1}H, ^{13}C and 2D NMR data of **DP5** in CD_3OD.

Position	Residue	$^{13}C^{a}$	$^{1}H^{a}$ (J in Hz)	^{1}H-^{1}H COSY	^{1}H-^{13}C HMBC
1	C	142.94	-		7.05, 7.64, 7.69
2	C	124.20	-		7.52, 7.53
3	CH	131.97	7.69, dd (7.8, 1.2)	7.53, 7.64	7.53, 7.64
4	CH	128.95	7.53, dt (8.7, 1.2)	7.52, 7.64, 7.69	7.52, 7.64
5	CH	132.48	7.64, dt (7.6, 1.4)	7.52, 7.53, 7.69	7.69
6	CH	131.74	7.52, d (7.6)	7.53, 7.64	7.53
7	C	139.66	-		7.23, 7.52
8/12	CH	130.22	7.05, d (8.2)	7.23	7.05, 7.23
9/11	CH	128.61	7.23, d (8.2)	7.05	7.05, 7.23
10	C	137.75	-		7.05
13	C	156.63	-		7.69
18	CH_2	45.59	5.19,		7.23
20	C	156.81	-		5.19

[a]Chemical shifts in ppm.
